# Highly sensitive and specific protein detection via combined capillary isoelectric focusing and proximity ligation

**DOI:** 10.1038/s41598-017-01516-7

**Published:** 2017-05-04

**Authors:** Narendra Padhan, Junhong Yan, Annegret Boge, Elaine Scrivener, Helgi Birgisson, Agata Zieba, Mats Gullberg, Masood Kamali-Moghaddam, Lena Claesson-Welsh, Ulf Landegren

**Affiliations:** 1 Uppsala University, Dept. of Immunology, Genetics and Pathology, Science for Life Laboratory, Biomedical Center, 751 08 Uppsala (JY, AZ, MKM, UL), Rudbeck Laboratory 751 85, Uppsala, (NP, LCW) Sweden; 2 ProteinSimple, 3001 Orchard Parkway, San Jose, California 95134 USA; 3 Uppsala University, Dept. of Surgical Sciences, Uppsala University Hospital, entrance 70, 751 85 Uppsala, Sweden; 4 Olink Bioscience, Dag Hammarskjölds väg 52B, 752 37 Uppsala, Sweden; 5 Department of Biomedical Engineering, Institute for Complex Molecular Systems, Eindhoven University of Technology, Eindhoven, 5600 MB The Netherlands

## Abstract

Detection and quantification of proteins and their post-translational modifications are crucial to decipher functions of complex protein networks in cell biology and medicine. Capillary isoelectric focusing together with antibody-based detection can resolve and identify proteins and their isoforms with modest sample input. However, insufficient sensitivity prevents detection of proteins present at low concentrations and antibody cross-reactivity results in unspecific detection that cannot be distinguished from bona fide protein isoforms. By using DNA-conjugated antibodies enhanced signals can be obtained via rolling circle amplification (RCA). Both sensitivity and specificity can be greatly improved in assays dependent on target recognition by pairs of antibodies using *in situ* proximity ligation assays (PLA). Here we applied these DNA-assisted RCA techniques in capillary isoelectric focusing to resolve endogenous signaling transducers and isoforms along vascular endothelial growth factor (VEGF) signaling pathways at concentrations too low to be detected in standard assays. We also demonstrate background rejection and enhanced specificity when protein detection depended on binding by pairs of antibodies using *in situ* PLA, compared to assays where each antibody preparation was used on its own.

## Introduction

Capillary electrophoresis (CE), coupled with fluorescence detection, liquid chromatography or mass spectrometry, has many applications in separating and detecting biomolecules such as DNA^1^, metabolites^2^, peptides^3^ and proteins^4^, since it offers high resolution and uses small amounts of samples. In particular, capillary isoelectric focusing (IEF) has proven useful for resolving protein isoforms such as phosphorylated variants^5^, since they typically have different isoelectric points (pI), allowing their separation in an ampholyte gradient gel.

The NanoPro 1000 system from ProteinSimple is a recently developed automated capillary IEF system where proteins are first separated and then immobilized in capillaries, followed by antibody probing in analogy to standard immunoblotting for protein identification and quantification^6^. However, the system differs from immunoblotting in two important respects^7^: First, in conventional immunoblotting proteins are separated according to molecular weight, while separation by IEF depends on the charge of the investigated protein species. Secondly, in immunoblotting, proteins are denatured by treatment with an ionic detergent while proteins separated by capillary IEF remain in a native state, which may influence the ability of antibodies to recognize the proteins. The capillary IEF method permits the use of limited tissue samples, as the technique efficiently resolves and quantifies proteins and their isoforms in submicroliter samples. Native proteins extracted from lysates of cells or tissues are separated according to charge, resolving isoforms of the proteins, whereupon the proteins are immobilized on the internal surface of the capillary wall through UV-mediated crosslinking. Proteins of interest are then detected using specific primary antibodies followed by horseradish peroxidase (HRP)-conjugated secondary antibodies, directed against the primary antibodies. The signal is generated by chemiluminescence and recorded as an electropherogram. The capillary instrument is attractive for studying protein phosphorylation in signaling pathways^8–10^, as the same antibodies can be used to provide quantitative information for both phosphorylated and unphosphorylated proteins, only small amounts of samples are required, and the entire assay is automated after sample preparation.

The signal strength of antibody-mediated detection assays can be enhanced by using antibodies with conjugated oligonucleotides that prime localized RCA of circularized DNA strands^11^. Another detection technique that uses oligonucleotide-conjugated antibodies, PLA, first reported in 2002, has been shown to improve both specificity and sensitivity of assays that use antibodies or other affinity reagents to detect proteins in a variety of matrices and states^12–14^. Pairs of oligonucleotide-conjugated antibodies that bind in proximity give rise to linear reporter DNA strands via DNA ligation, which can then be amplified by PCR for sensitive detection. The requirement for binding by pairs of antibodies also provides a means to enhance specificity of detection over single-binder assays by ignoring any cross-reactivity for irrelevant proteins that is not shared by the two primary antibodies used in the assay. *In situ* PLA is a variant of the PLA technique, first described by Söderberg *et al*. in 2006, where pairs of antibody-DNA conjugates recognize the same or pairs of interacting proteins of interest and then direct the formation of a circular reporter DNA strand^15^. This DNA circle is locally amplified via RCA, providing excellent specificity and sensitivity of detection in microscopy^15^ and flow cytometry^16^. Moreover, Liu *et al*. described improved sensitivity and specificity of protein detection in immunoblotting onto membranes via *in situ* PLA^17^. Capillary IEF provides an attractive opportunity to apply *in situ* PLA in an automated system using minimal amounts of reagents, and we here used this strategy for sensitive detection of proteins of relevance for angiogenesis in order to enhance specificity of protein detection via dual recognition.

The angiogenic process, which results in the development of new blood vessels, plays important roles in the progression of cancer from small, localized neoplasms to larger, potentially metastatic tumors^18^. Vascular endothelial growth factor A (VEGFA) mediates this process mainly through binding to VEGF receptor 2, leading to downstream signal transduction, which involves phosphorylation of signal transducers regulating various short and long-term biological responses^19^. The state of activity of this signaling pathway can be monitored by studying the phosphorylation of the signal transducing proteins that are present at low concentration in e.g. tissue culture cell lysates or scarce tumor biopsy samples. Accordingly, these analyses exemplify the need for assays with minimal sample consumption and excellent specificity for the targeted proteins, provided by the combination of capillary IEF with RCA and *in situ* PLA.

## Results

### Increased sensitivity in detection of ERK by NanoPro+RCA

**Figure 1:**
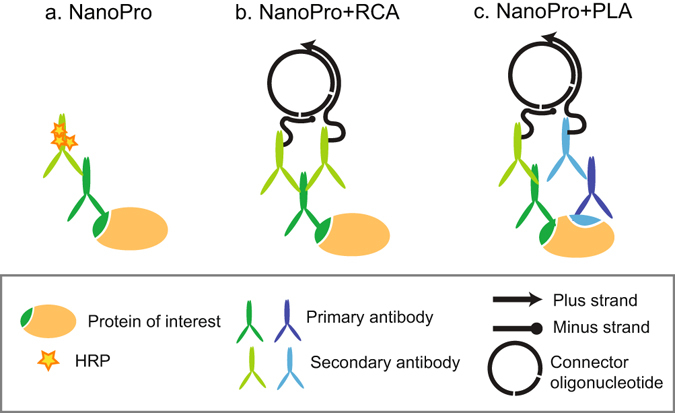
Schematic representation of different assay formats used in the study. (**a**) Capillary IEF using a standard NanoPro 1000 assay. (**b**) RCA-enhanced NanoPro 1000 assay. (**c**) PLA-enhanced NanoPro 1000 assay.

**Figure 2:**
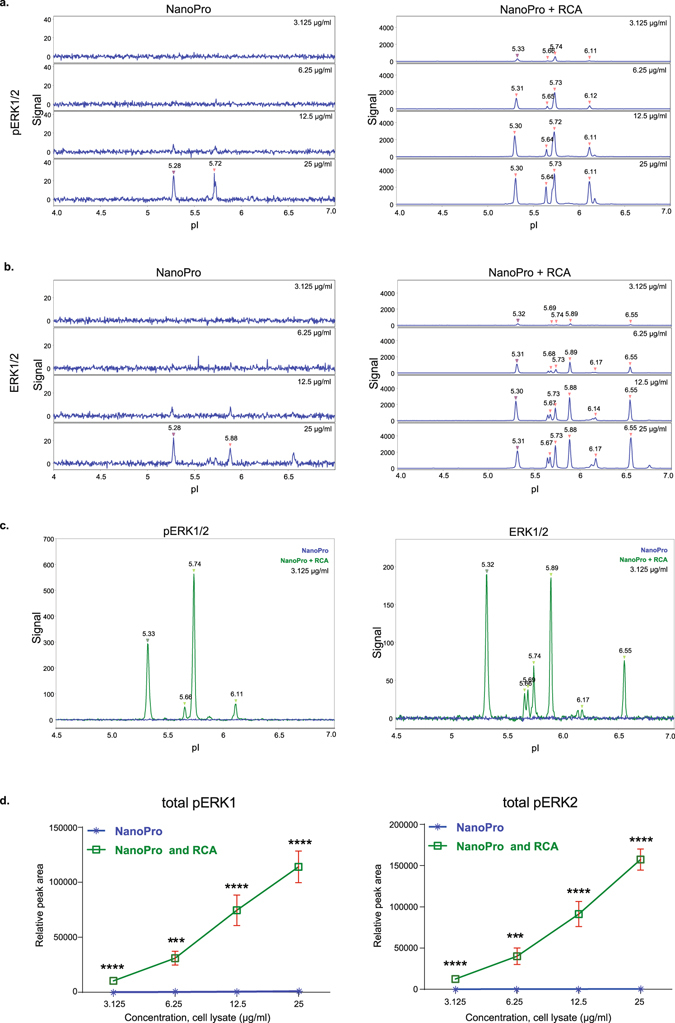
Sensitive detection of phosphorylated and total ERK1/2 protein. (**a**) Electropherogram of phospho-ERK1/2 (pERK1/2) from serially diluted HUVEC lysate (25 µg/ml to 3.125 µg/ml), using the standard NanoPro assay (left) and NanoPro+RCA (right). (**b**) Detection of ERK1/2 as in (**a**). (**c**) Detailed overlay graph of pERK1/2 (left) and ERK1/2 (right) analyzed by standard NanoPro assay (blue) and NanoPro+RCA (green) in 3.125 µg/ml cell lysate. (**d**) Areas extracted from pI peaks corresponding to total pERK1 and pERK2 (as in **c**), from 25 µg/ml to 3.12 µg/ml serially diluted cell lysates. Quantification was done from three biological replicates (n = 3) with three technical replicates for each sample. Statistical significances were calculated using Student’s unpaired t-tests. p < 0.05 was considered statistically significant. ***p < 0.001 and ****p < 0.0001. Error bars in the graph represent mean ± SEM.

The purpose of this study was to investigate if protein detection by capillary IEF could be enhanced by molecular genetic means involving RCA (Fig. 1). In a first approach, a standard NanoPro 1000 assay with detection by a single primary antibody (Fig. 1a) was combined with ligation and amplification steps (NanoPro+RCA, Fig. 1b). Thus, in the NanoPro+RCA procedure, a pair of secondary antibodies conjugated with DNA oligonucleotides was used instead of HRP-labeled secondary antibodies as in the standard NanoPro assay. The method was used to detect both unphosphorylated and phosphorylated extracellular regulated kinase 1/2 (ERK1/2 and pERK1/2), using the same primary antibody preparation. The pERK1/2 and total ERK1/2 antibodies were validated using conventional immunoblotting (Fig. S1). Lysates from cultured human umbilical vein endothelial cells (HUVEC) treated with VEGFA were analyzed by capillary isoelectric focusing at dilutions from 25 µg/ml to 3.125 µg/ml (corresponding to 10 -1.25 ng protein mass). As shown in Fig. 2a (left) NanoPro-detection of pERK1/2 allowed visualization of the pI 5.28 and 5.72 peaks only at the highest lysate concentration, yielding a poor signal/noise (S/N) ratio. Detection with NanoPro+RCA, by contrast, allowed detection of pERK1/2 already at 3.125 µg/ml, equal to an at least 8-fold lower limit-of-detection (Fig. 2a, right). A similar fold-increase in sensitivity was observed when analyzing total ERK1/2 proteins in the endothelial cell lysate (Fig. 2b). Figure 2c compares the detection of the various ERK1/2 phosphoforms and total protein forms in VEGFA-treated endothelial cells at high resolution. The relative peak area of total pERK1 (Fig. 2d, left) and total pERK2 (Fig. 2d, right) forms, increased with increasing protein concentration. These data demonstrate the greatly enhanced signal strength, and thus the reduced sample requirement, using NanoPro+RCA compared to the standard technique.

### Detection of VEGFA-induced signal transducers by NanoPro+RCA

**Figure 3:**
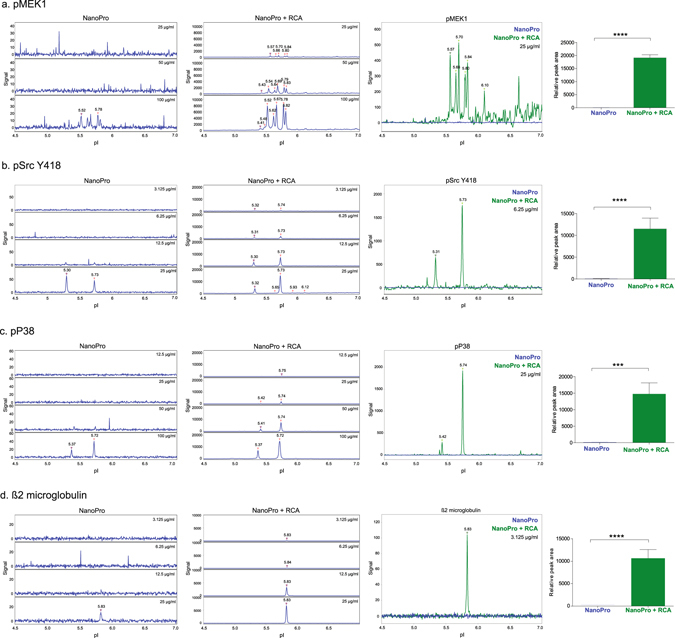
Detection of VEGFA-induced signal transducers. (**a–d**) Lysate of HUVECs treated with VEGFA and analyzed for pMEK1 (**a**), pSrc Y418 (**b**), pP38 (**c**), ß2 microglobulin (**d**) were detected with standard NanoPro assays (left panel) or NanoPro+RCA (middle panel). Detailed overlay graphs of protein detection with standard NanoPro assay (blue) and NanoPro+RCA (green) are shown in the right panel. Quantifications were done from three biological replicates (n = 3) with 4 technical replicates in each sample. Statistical significances were calculated using Student’s unpaired t-tests. p < 0.05 was considered statistically significant. ***p < 0.001 and ****p < 0.0001. Error bars in the graph represent mean ± SEM.

Several signal transducers phosphorylated in response to VEGFA treatment of HUVECs endothelial cells were detected with considerably increased sensitivity by combining NanoPro-assays with enhanced detection via RCA. Thus, phosphorylated mitogen activated protein kinase kinase 1 (pMEK1) forms were barely detectable by NanoPro alone at a lysate concentration of 100 µg/ml whereas NanoPro+RCA revealed a reproducible pattern of peaks at 25 µg/ml (Fig. 3a, compare left and middle panels; the right panel illustrates the high-resolution pattern and quantification of pMEK1 peaks). Cytoplasmic tyrosine kinase Src phosphorylated on the activating tyrosine 418 (pSrc Y418; Fig. 3b) and phosphorylated p38 mitogen activated protein kinase (pP38MAPK) (Fig. 3c), were also reproducibly detected by NanoPro+RCA at much lower lysate concentrations than by standard NanoPro. Figure 3d shows the detection of β2 microglobulin, conventionally used as a loading control for normalization between samples, using NanoPro alone or combined NanoPro+RCA. As a control, we used the same antibodies also in unstimulated cell lysates to confirm that the peaks in the electropherogram shown in Fig. 3 indeed were induced in response to VEGFA stimulation (Fig. S2). NanoPro1000+RCA was further used to detect the total MEK1/2 and Src protein pools with several-fold increased sensitivity compared to detection by NanoPro1000 alone (Fig. S3). However, none of several commercial antibodies tested allowed detection of total P38MAPK by NanoPro. For validation of the phosphorylated and total protein antibodies against MEK1, pSrc Y418 and P38MAPK, as well as β2 microglobulin by conventional immunoblotting, see Fig.﻿﻿ S1.

### Sensitive detection of signaling in human cancer samples

**Figure 4:**
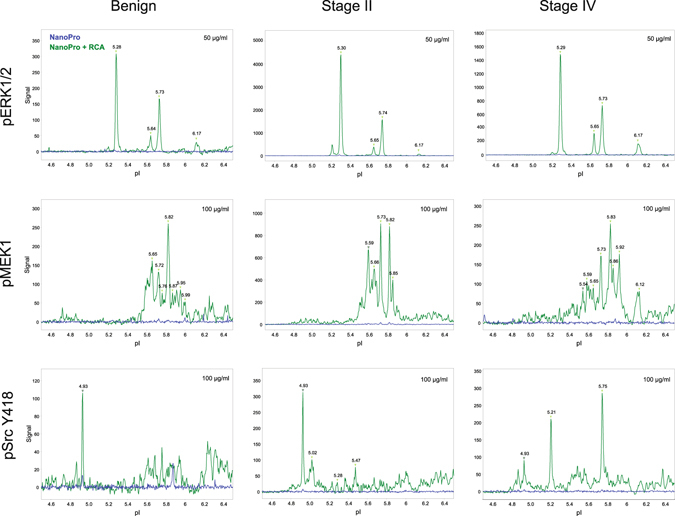
Detection of signal transducers in colorectal cancer samples. Visualization of pERK1/2 (top), pMEK1 (middle) and pSrc Y418 (bottom) in human colorectal cancer samples from patients at different stages of the disease. Detailed overlay graphs of protein detection with standard NanoPro assay (blue) and NanoPro+RCA (green) are shown.

To illustrate the application of the sensitive NanoPro+RCA technique to normal colorectal mucosa and cancer specimens from patients at distinct stages of the disease, signal transducers pERK1/2, pMEK1 and pSrc Y418 were analyzed in human tissue samples. Benign colorectal tissue as well as stage II and stage IV colorectal cancer tissues, were analyzed by NanoPro alone or by NanPro+RCA (Fig. 4). Whereas detection by NanoPro alone failed at 50–100 µg/ml concentrations of tissue lysates (equals to 20–40 ng of protein mass), NanoPro+RCA allowed sensitive detection in all samples of pERK1/2 (Fig. 4 top panels), pMEK1 (Fig. 4 middle panels) and pSrc Y418 (Fig. 4 bottom panels), demonstrating the ability to reduce the amount of hard-to-come by samples via the RCA-assisted detection reaction. Analysis of HUVEC samples using total protein antibodies revealed both overlapping peaks and unique peaks (Fig. S4), where unique peaks occasionally were detected by antibodies reacting with the phosphoproteins, but not by the total protein antibodies due to the relatively low abundance of the phosphoproteins. In agreement, we have previously demonstrated that the pSrc Y418 antibodies detect two peaks P1 and P2 with more acidic pI than peaks P3 and P4 detected by the total protein Src antibody in human endothelial cells^20^.

### Improved specificity by dual recognition NanoPro+PLA

In traditional protein immuno assays like immunoblots or standard NanoPro assays, a single primary antibody preparation directed against the protein of interest is applied to the sample, followed by secondary antibody modified for detection. The specificity of these assays thus relies on the specificity of the primary antibody preparation. If the primary antibody has unspecific affinity towards proteins unrelated to the protein of interest, multiple bands or peaks may appear, as is commonly seen. This situation can be greatly mitigated if detection depends on binding by two primary antibodies as the chance that they both recognize the same unspecific protein is low. The mechanism of dual recognition has been available for antibody-assisted detection of protein in solution phase since the 1960s via sandwich assays, offering greatly improved specificity over single-binder assays^21^. *In situ* PLA offers an attractive opportunity to render localized signal amplification via RCA dependent on binding by not just one but two distinct primary antibodies, for improved specificity of detection. To investigate this mechanism with capillary IEF, mouse monoclonal antibodies and rabbit polyclonal antibodies, raised against the same target proteins, were applied separately in a standard NanoPro analysis or as NanoPro+RCA analyses using HUVEC lysates as described above. The results were compared to those where target binding by both mouse and rabbit antibodies were required for detection by combining NanoPro with *in situ* PLA (NanoPro+PLA, Fig. 1c).

**Figure 5:**
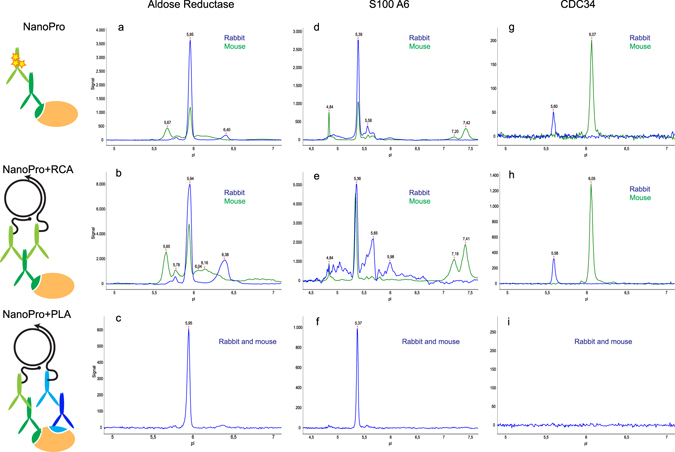
Detection of proteins by antibody pairs in NanoPro+PLA. Aldose reductase (**a–c**), S100A6 (panels **d–f**) and CDC34 (panels **g–i**) were detected in 50 µg/ml HDMEC cell lysates using different protocols. In (**a**,**d** and **g**), the standard NanoPro assay was used with monoclonal mouse antibodies (blue) or rabbit affinity purified polyclonal antibodies (green). Panels (**b,e** and **h**) display results when the same antibodies were applied in NanoPro+RCA. Panels (**e,f** and **i**), illustrate results when recognition depended on binding by both the mouse and rabbit antibodies in NanoPro+PLA. Schematic representation of different assay formats, are shown left of the panels.

Detection of aldose reductase in endothelial cell lysates by NanoPro using mouse monoclonal antibodies alone resulted in a major peak of pI 5.9, and in addition several minor peaks appeared. Detection with rabbit monoclonal antibodies also resulted in a major peak at pI 5.9, along with several minor peaks, distinct from those revealed by the mouse antibodies (Fig. 5a). As expected NanoPro+RCA identified similar peaks as those seen with standard NanoPro detection for the primary mouse and rabbit antibodies, although with slightly different profiles (Fig. 5b). In contrast, when detection relied on coincident recognition by the two primary antibodies using NanoPro+PLA detection, a sole distinct peak at pI 5.9 was recorded (Fig. 5c). A similar effect was seen when analyzing S100A6 in the same endothelial cell lysates. Both mouse and rabbit primary antibodies applied for standard NanoPro analysis (Fig. 5d) or NanoPro+RCA (Fig. 5e), resulted in a main peak of pI around 5.3, but with several additional peaks differentially detected by the two antibody preparations. Using NanoPro+PLA only a single peak of pI 5.37 appeared, which was recognized by both antibody preparations (Fig. 5f).

The increased specificity using dual antibody recognition was further demonstrated by the complete loss of detection signals when a pair of primary antibody preparations raised against CDC34 was used for NanoPro+PLA. In this case, each of the rabbit and mouse antibody preparations detected distinct peaks at pI 5.5 and pI 6.0, respectively (compare Fig. 5**g–i**). The analysis strongly suggests that at least one, but maybe both of the peaks identified by the two antibody preparations are nonspecific in the relatively native conditions of isoelectric focusing. Immunoblotting analyses of SDS-treated samples confirmed the expected molecular masses for aldose reductase and S100A6, while the two CDC34 antibody preparations detected several other bands of both higher and lower apparent molecular mass in addition to the expected 34 kDa band (Fig. S5).

## Discussion

Improved sensitivity and specificity of detection of proteins and their isoforms are central aims in proteomics research, because of the complexity of biological specimens, and the need to also detect proteins present in minute concentrations. DNA-assisted technologies for protein detection such as proximity ligation assays can offer improved sensitivity of detection via DNA amplification, and the specificity may be enhanced over assays that depend on binding by single antibodies, by virtue of the requirement for joint target protein detection by two or more antibodies in PLA. *In situ* PLA, applied on traditional immunoblotting membranes, has been shown to improve detection specificity through the use of pairs of antibodies, as well as detection sensitivity through the use of RCA^17^. However, *in situ* PLA requires more handling than conventional immunoblots, and relatively large amounts of expensive reagents are needed.

The application of capillary IEF to study protein phosphorylation on signaling pathway has been demonstrated in studies of mouse tumor tissue^22^, human tumor xenografts on mouse^8^ and human tumor tissue^23, 24^. Here, we could show great signal increase by RCA-assisted reactions, compared to standard assay using the same primary antibodies. We titrated different parameters for use with the capillary IEF system (Figs S6–S8), and found that probe concentrations and the duration of RCA were both positively related to the signal intensity (Fig. S7). RCA-mediated detection resulted in an increased sensitivity so that distinct peaks, representing components in the VEGFA signaling cascade, could still be resolved at concentrations of cell lysates where peaks were no longer detected using the standard NanoPro assay (Fig. 3). This enhanced sensitivity was also observed for tumor tissue lysates (Fig. 4).

Apart from matters of sensitivity, another important requirement in affinity-based protein analysis methods is the reliability of correct target detection by the antibodies used. Both mono- and polyclonal antibody preparations frequently exhibit cross-reactivity for irrelevant target molecules, and this becomes particularly troubling when the cross-reactive targets are present in high concentrations relative to the proper target molecules. We exemplified in the capillary IEF system that mouse and rabbit antibody preparations, raised against the same target proteins, can give rise to different profiles. The problem of unspecific detection is well known in immunoblotting as reflected in multiple bands that may correspond to some combination of correct and irrelevant target proteins. This renders results difficult to interpret and may preclude correct identification of peaks against a noisy background. We demonstrate that by combining pairs of independent antibody preparations, raised against the same proteins, using *in situ* PLA, risks of false detection signals can be greatly reduced (Fig. 5). The fact that the PLA signal indeed depends on both primary antibodies binding the same protein, is clearly illustrated by a case where the primary mouse and rabbit antibodies against CDC34 showed single, nonoverlapping peaks using NanoPro and NanoPro+RCA. In this case no peaks were detected using the NanoPro+PLA assay format (Fig. 5g–i). It is unclear if the peaks revealed by these antibodies correspond to the correct CDC34 protein or if they represent unspecific reactivity. We note that a theoretical calculation of the pI for CDC34 in its unphosphorylated state is 4.41^25^. Moreover, immunoblotting with the CDC34 antibodies required long exposure that eventually revealed multiple molecular species in addition to the expected 34 kDa components (Fig. S5).

In cases such as those demonstrated herein where the two primary antibody preparations were derived from distinct species, standard secondary antibody-DNA conjugates can be used for *in situ* PLA. Alternatively, primary antibodies can also be directly conjugated to DNA strands allowing primary antibodies from the same species to be used for protein and isoform detection (Fig. S6 and Supporting information).

In conclusion, we demonstrate improved sensitivity of detection using RCA combined with immunoprobing in capillary IEF, and thus protein and isoforms can be detected that are present at very low concentrations or in minimal sample aliquots. We also illustrate that PLA can greatly improve specificity of detection because of the requirement that target molecules are simultaneously recognized by two primary antibodies, even when these antibodies individually exhibit cross-reactivity for more protein species.

## Methods

### Cell culture, stimulation and lysis

Human umbilical vein endothelial cells (HUVECs; ATCC) and human dermal microvascular endothelial cells (HDMECs; ATCC) were cultured on gelatin-coated 10 cm tissue culture petri dishes in endothelial cell basal medium MV 2 (EBM-2, C-22221; PromoCell) with supplemental pack C-39221, containing 5% fetal calf serum, epidermal growth factor (5 ng/ml), basic fibroblast growth factor (10 ng/ml), insulin-like growth factor-1 (20 ng/ml), vascular endothelial growth factor 165 (0.5 ng/ml) ascorbic acid (1 μg/ml) and hydrocortisone (0.2 μg/ml). HUVECs/HDMECs at passages 3–6 were used. For experimental purposes, HUVECs and HDMECs were serum-starved overnight and plated in EBM-2 medium, 0.5% FCS without growth factor supplement and treated with/without VEGF (50 ng/ml) for 7 min. HUVECs/HDMECs were lysed in Bicine/CHAPS buffer containing DMSO inhibitor mix (#040-510, ProteinSimple) and aqueous inhibitor mix (#040-482, ProteinSimple). The cell lysates were clarified by centrifugation and protein concentrations were determined by using BCA Protein Assay Kit (Pierce, Rockford, IL, USA).

### Tumor biopsy collection and lysis

The colorectal tumor sampling and characterization of the anonymous samples were both approved by the Uppsala Regional Ethical Review Board (no 2007/005 and 2000/001) and they were carried out in accordance with the relevant guidelines. Prior to the operation the patients were asked by the responsible surgeon if they would donate tumor tissues and blood samples for future molecular studies. The patients were given written study information and signed an informed consent form for the storage, isolation of protein, and use of the material in research projects if they accepted to participate. When the surgical specimens (colon tissue) had been removed from the patients they were transported on ice to the histopathological department and a clinical pathologist cut a 5 × 5 × 5 mm biopsy from the periphery of the primary tumor and 10 × 10 mm normal mucosa more than 5 cm from the primary tumor. The biopsies were immediately placed dry in test tubes and kept in a −80 °C freezer until analyzes were made. The colon cancer samples or normal mucosa were selected from a set of frozen tumor biopsies from patients operated upon for colorectal cancer at the hospitals in Karlstad or Västerås, Sweden. Tissue samples were homogenized and lysed in RIPA buffer containing phosphatase and protease inhibitors (ProteinSimple, San Jose, CA). The tissue lysates were clarified by centrifugation and protein concentration was measured by using BCA Protein Assay Kit (Pierce, Rockford, IL, USA).

### Antibodies and antibody-DNA conjugates

All antibodies used in this study were validated by conventional immunoblotting (see Figs S1 and S5). See Table S1 for antibody source and dilution used for detection in the NanoPro1000 with and without RCA/PLA and by immunoblotting. Recombinant human VEGFA (293-VE-010) was purchased from R&D Systems.

Horseradish peroxidase (HRP) conjugated anti-mouse and anti-rabbit antibodies were from ProteinSimple (#040-655, #040-656). The following secondary antibody-DNA conjugates were provided by Olink for Duolink *In Situ* PLA (the reagents are now available from Sigma Aldrich): Probe Anti-Mouse PLUS (Affinity Purified Donkey Anti-Mouse IgG, H+L; DUO92001), Duolink *In Situ* PLA Probe Anti-Rabbit PLUS (Affinity Purified Donkey Anti-Rabbit IgG, H+L; DUO92002), Duolink *In Situ* PLA Probe Anti-Goat PLUS (Affinity Purified Donkey Anti-Goat IgG, H+L; DUO92003), Duolink *In Situ* PLA Probe Anti-Mouse MINUS (Affinity Purified Donkey Anti-Mouse IgG, H+L; DUO92004), Duolink *In Situ* PLA Probe Anti-Rabbit MINUS (Affinity Purified Donkey anti-Rabbit IgG, H+L; DUO92005), Duolink *In Situ* PLA Probe Anti-Goat MINUS (Affinity Purified Donkey Anti-Goat IgG, H+L; DUO92006).

### Protein detection by standard NanoPro assays

Cell lysates were diluted in Bicine/CHAPS Lysis Buffer and Sample Diluent (#040-764, ProteinSimple) and mixed with either Premix G2, pH 3–10 separation gradient (#040-968, ProteinSimple) with fluorescence labeled pI Standard Ladder 1 (#040-644, ProteinSimple), or Premix G2, pH 5–8 separation gradient (#040-972, ProteinSimple) with fluorescence labeled pI Standard Ladder 3 (#040-646, ProteinSimple), for analysis on the NanoPro1000 system (ProteinSimple) according to the manufacturer’s instructions. Capillary isoelectric focusing electrophoresis was carried out at 21 mW for 40 minutes. The separated proteins were immobilized on the capillary walls by exposure to UV light for 100 seconds using instrument default settings. The washing step was set for 20 sec wash load time and 150 sec wash soak time. Immunoprobing was carried out by first incubating with primary antibodies, diluted in Antibody Diluent (#040-309, ProteinSimple) for 2 hrs, followed by 2 washes. The capillaries were then incubated with HRP-labeled secondary antibodies for 1 hr, followed by two washes. A 1:1 mixture of Luminol and Peroxide XDR (#040-652, #041-084, ProteinSimple) was then added to generate chemiluminescent light, which was captured by a charge-coupled device camera with 6 exposure times (30, 60, 120, 240, 480 and 960 sec). The digital images were analyzed and quantified with Compass software (ProteinSimple), according to the manufacturer’s instructions. The Compass analysis extracts signal to noise (S/N) data at given pI values in the plots.

### Protein detection by NanoPro with RCA and NanoPro with PLA

Protein separation and immobilization in capillaries followed the standard protocol for the instrument. For NanoPro with RCA (NanoPro+RCA), a modified immuno RCA protocol was used where single primary antibodies were detected by pairs of antibodies with conjugated oligonucleotides that were used to template the formation of a DNA circle and priming of RCA. The immunoprobing step started by the addition of target-specific primary antibodies. Next, a pair of secondary antibody-DNA conjugates (PLUS and MINUS, Olink), both directed against the species of the primary antibodies were diluted 5-fold in PLA buffer (Olink) and incubated in the capillary for 1 hr, followed by two washes.

For NanoPro with PLA (NanoPro+PLA), mouse and rabbit primary antibodies directed against the same protein analyte were applied to the capillary and incubated for 1 hr, followed by two washes. Then a pair of secondary antibody-DNA conjugates (anti-mouse PLUS and anti-rabbit MINUS, or anti-rabbit PLUS and anti-mouse MINUS Olink) were incubated for 1 hr and followed by 2 washes. After immunoprobing for single- (NanoPro+RCA) or double-binder PLA (NanoPro+PLA), a ligation mix (Olink) was added to the capillaries for 30 min, followed by two washes. Then the RCA step was carried out by Duolink® *In Situ* Detection Reagents Brightfield (DUO92012, Olink) for either 2 or 4 hrs. After two washes, the capillaries were incubated with detection oligonucleotides (DUO92012, Olink) for 1 hr. The detection step using Luminol and Peroxide XDR and image acquisition are the same as in the standard capillary IEF assay. The washing steps were set for 20 sec wash load time and 150 sec wash soak time.

### Immunoblotting

Ten μg of HUVEC lysates were mixed with LDS sample buffer and Sample Reducing Agent (Life Technologies) heated at 70 °C for 10 min. The proteins were resolved by NuPAGE Novex 4–12% Bis-Tris SDS PAGE Gel (Life Technologies) and transferred onto PVDF membranes (Immobilon-P IPVH00010, Millipore), blocked by 5% (w/v) nonfat dry milk/BSA in TBS with 0.1% Tween 20 for 1 h at RT followed by incubation over night at 4 °C with primary antibodies. Proteins of interest were detected with Rabbit IgG, HRP-linked whole Ab (from donkey) #NA934 or Mouse IgG, HRP-linked whole Ab (from sheep) #NA931, in 1: 15000 dilutions from GE Healthcare and visualized with using ECL Prime (#RPN2232, GE Healthcare) and exposed to ChemiDocTM MP Imaging System (Bio-Rad Laboratories). See Table S1 for antibody source and working dilutions.

## Electronic supplementary material

Supplementary information

## References

[1] Swerdlow H, Gesteland R (1990). Capillary gel electrophoresis for rapid, high resolution DNA sequencing. Nucleic Acids Res.

[2] Roscher J (2014). Nonaqueous capillary electrophoresis as separation technique to support metabolism studies by means of electrochemistry and mass spectrometry. Electrophoresis.

[3] Ali I, Al-Othman ZA, Al-Warthan A, Asnin L, Chudinov A (2014). Advances in chiral separations of small peptides by capillary electrophoresis and chromatography. J Sep Sci.

[4] Biacchi M (2014). Analysis of monoclonal antibody by a novel CE-UV/MALDI-MS interface. Electrophoresis.

[5] Shiraishi M, Loutzenhiser RD, Walsh MP (2005). A highly sensitive method for quantification of myosin light chain phosphorylation by capillary isoelectric focusing with laser-induced fluorescence detection. Electrophoresis.

[6] O’Neill RA (2006). Isoelectric focusing technology quantifies protein signaling in 25 cells. Proc Natl Acad Sci USA.

[7] Burnette WN (1981). “Western blotting”: electrophoretic transfer of proteins from sodium dodecyl sulfate–polyacrylamide gels to unmodified nitrocellulose and radiographic detection with antibody and radioiodinated protein A. Anal Biochem.

[8] Chen JQ (2013). Capillary isoelectric-focusing immunoassays to study dynamic oncoprotein phosphorylation and drug response to targeted therapies in non-small cell lung cancer. Mol Cancer Ther.

[9] Iacovides DC (2013). Identification and quantification of AKT isoforms and phosphoforms in breast cancer using a novel nanofluidic immunoassay. Mol Cell Proteomics.

[10] Sabnis H, Bradley HL, Bunting ST, Cooper TM, Bunting KD (2014). Capillary nano-immunoassay for Akt 1/2/3 and 4EBP1 phosphorylation in acute myeloid leukemia. J Transl Med.

[11] Schweitzer B (2000). Immunoassays with rolling circle DNA amplification: a versatile platform for ultrasensitive antigen detection. Proc Natl Acad Sci USA.

[12] Fredriksson S (2002). Protein detection using proximity-dependent DNA ligation assays. Nat Biotechnol.

[13] Gullberg M (2004). Cytokine detection by antibody-based proximity ligation. Proc Natl Acad Sci USA.

[14] Darmanis S (2010). Sensitive plasma protein analysis by microparticle-based proximity ligation assays. Mol Cell Proteomics.

[15] Soderberg O (2006). Direct observation of individual endogenous protein complexes *in situ* by proximity ligation. Nat Methods.

[16] Leuchowius KJ, Weibrecht I, Landegren U, Gedda L, Soderberg O (2009). Flow cytometric *in situ* proximity ligation analyses of protein interactions and post-translational modification of the epidermal growth factor receptor family. Cytometry A.

[17] Liu, Y. *et al*. Western blotting via proximity ligation for high performance protein analysis. *Mol Cell Proteomics* **10**, O111 011031 (2011). 10.1074/mcp.O111.011031 PMC3226413 21813417

[18] Hanahan D, Weinberg RA (2011). Hallmarks of cancer: the next generation. Cell.

[19] Simons M, Gordon E, Claesson-Welsh L (2016). Mechanisms and regulation of endothelial VEGF receptor signalling. Nat Rev Mol Cell Biol.

[20] Sun Z (2012). VEGFR2 induces c-Src signaling and vascular permeability *in vivo* via the adaptor protein TSAd. J Exp Med.

[21] Wide L, Bennich H, Johansson SG (1967). Diagnosis of allergy by an *in-vitro* test for allergen antibodies. Lancet.

[22] Urasaki Y, Pizzorno G, Le TT (2016). Chronic Uridine Administration Induces Fatty Liver and Pre-Diabetic Conditions in Mice. PLoS One.

[23] Unger FT (2016). Nanoproteomic analysis of ischemia-dependent changes in signaling protein phosphorylation in colorectal normal and cancer tissue. J Transl Med.

[24] Padhan N (2016). High sensitivity isoelectric focusing to establish a signaling biomarker for the diagnosis of human colorectal cancer. BMC Cancer.

[25] Hornbeck PV (2015). PhosphoSitePlus, 2014: mutations, PTMs and recalibrations. Nucleic Acids Res.

