# Supplementary material for: Highly sensitive and specific protein detection via combined capillary isoelectric focusing and proximity ligation
Source: Sci Rep. 2017 May 4;7:1490. doi: 10.1038/s41598-017-01516-7 (PMC5431457; doi:10.1038/s41598-017-01516-7)
Supplement: Supplementary file 1 — Supplementary information [file 41598_2017_1516_MOESM1_ESM.doc]

**Supporting information**

Highly sensitive and specific protein detection via combined capillary isoelectric focusing and proximity ligation

Narendra Padhan^1+^, Junhong Yan^1,5*+^ , Annegret Boge^2^, Elaine Scrivener^2^, Helgi Birgisson^3^, Agata Zieba^1^, Mats Gullberg^3^, Masood Kamali-Moghaddam^1^, Lena Claesson-Welsh^1+^, Ulf Landegren^1*+^

1) Uppsala University, Dept. of Immunology, Genetics and Pathology, Science for Life Laboratory, Biomedical Center, 751 22 Uppsala (JY, AZ, MKM, UL), Rudbeck Laboratory 751 85 Uppsala (NP, LCW), Sweden

2) ProteinSimple, 3001 Orchard Parkway, San Jose, California, 95134 USA

3) Uppsala University, Dept. of Surgical Sciences, Uppsala University, Rudbeck Laboratory 751 85 Uppsala, Sweden

4) Olink Biosciences, Dag Hammarskjölds väg 52B, 752 37 Uppsala, Sweden

5) Current address: Department of Biomedical Engineering, Institute for Complex Molecular Systems, Eindhoven University of Technology, Eindhoven 5600 MB, The Netherlands

^+^Shared first and last authorships

*Corresponding authors: unhongyan826@gmail.com, [ulf.landegren@igp.uu.se](mailto:ulf.landegren@igp.uu.se)

**
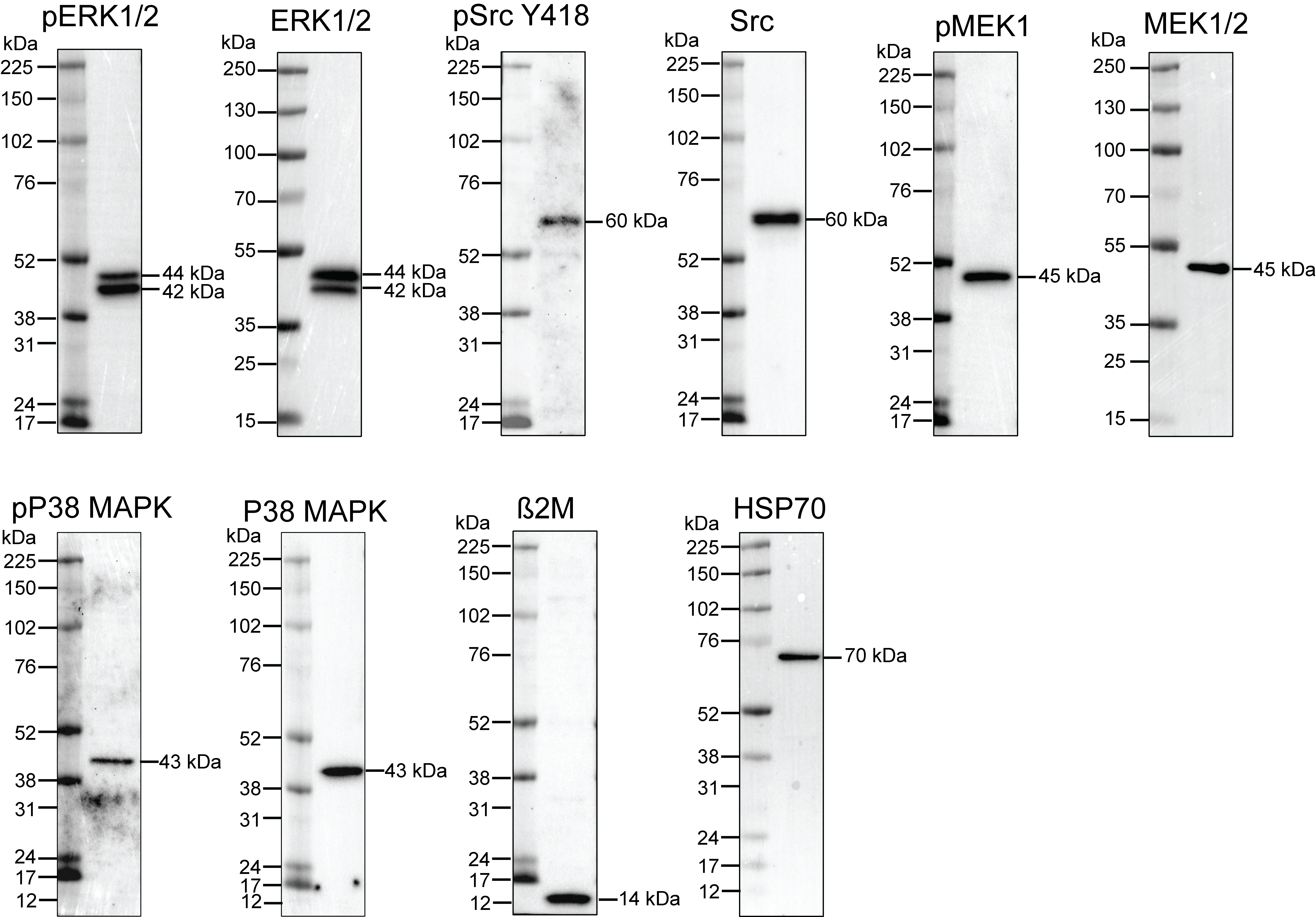
**

**Figure S1.** Antibody validation. All antibodies used for isoelectric focusing in main Figure 2 and 3 were verified by immunoblotting. For each blot two images, colorimetric and chemiluminescent, were acquired by a ChemiDoc™ MP Imaging System (Bio-Rad). The images were merged and shown here to assess the molecular size and specificity of each antibody. Ten µg of HUVEC cell lysate was used in each blot. Molecular weight markers (225-12 kDa or 250-15 kDa) run in parallel are indicated to the left.


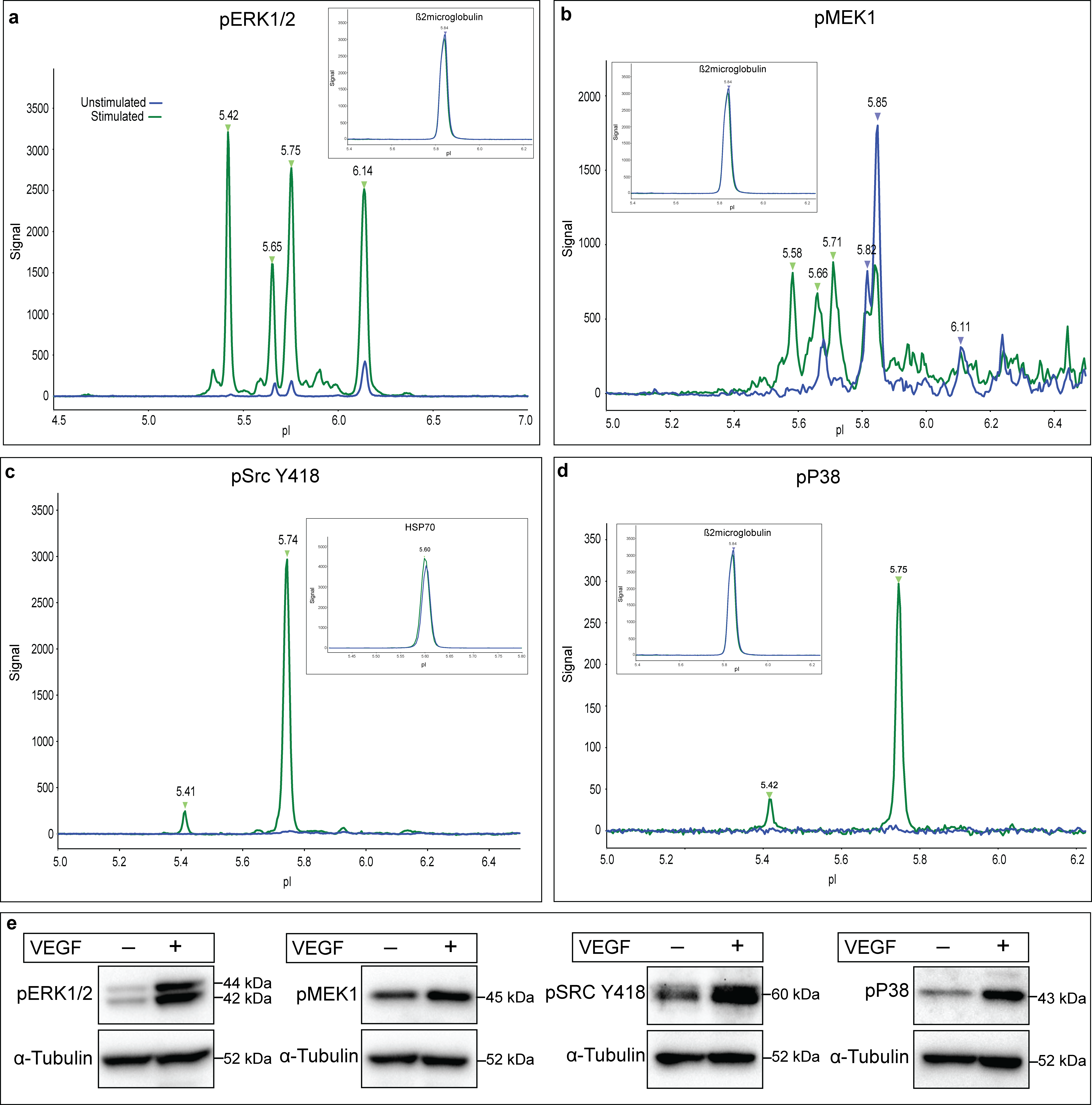


**Figure S2.** Detection of signal transducers in human umbilical vein endothelial cell (HUVEC) lysates with NanoPro+RCA. Visualization of pERK1/2 (a), pMEK1 (b), pSrc Y418 (c) and pP38 (d) in HUVEC lysates after treatment of cells with or without 50 ng/ml VEGFA. Detailed overlay graphs of protein detection with NanoPro+RCA are shown for HUVEC lysates with (green) or without (blue) stimulation with VEGFA. Graphs of loading control proteins (β2 microglobulin and heatshock protein 70; HSP 70) are shown as insets in each panel. e. Immunoblot validation of unstimulated (without VEGF) and stimulated (with VEGF) HUVEC lysates used for NanoPro+RCA. α-Tubulin was used as loading control in the immunoblots. Data complement those shown in main Figures 2, 3 and 4.

**
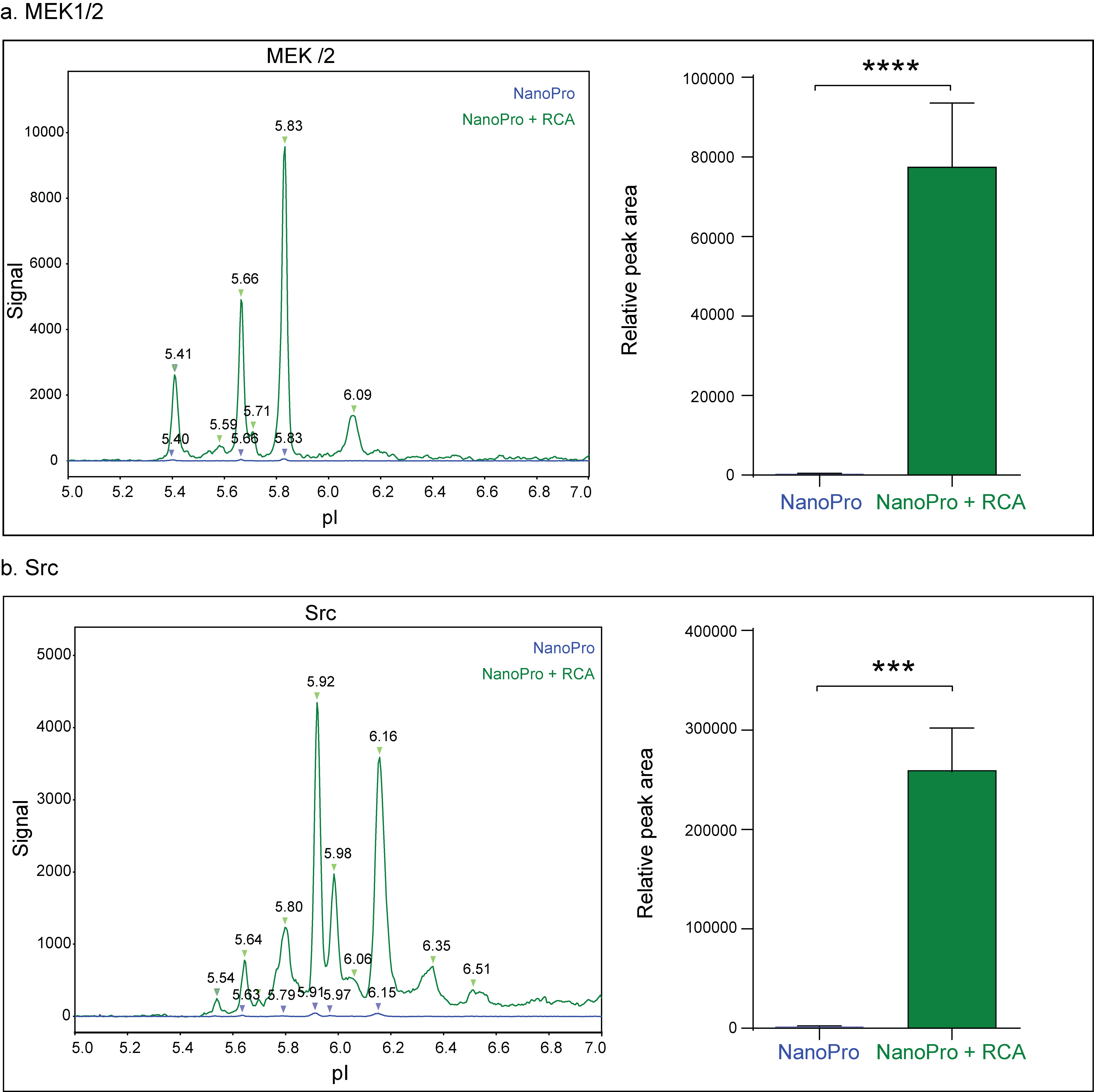
**

**Figure S3**. Detection of total MEK1/2 and Src protein. Electropherogram (left) and quantification (right) of MEK1/2 (a) and Src (b) in HUVEC cell lysates, 25 µg/ml and 50 µg/ml respectively. The standard NanoPro assay is shown as blue peaks and NanoPro+RCA as green peaks. Quantification were done from three biological replicates (n=3) with two-four technical replicates in each sample. Statistical significances were calculated using Student’s unpaired t-tests. p < 0.05 was considered statistically significant. ***, p < 0.001 and ****, p < 0.0001. Error bars in the graph represents mean ± SEM.

**
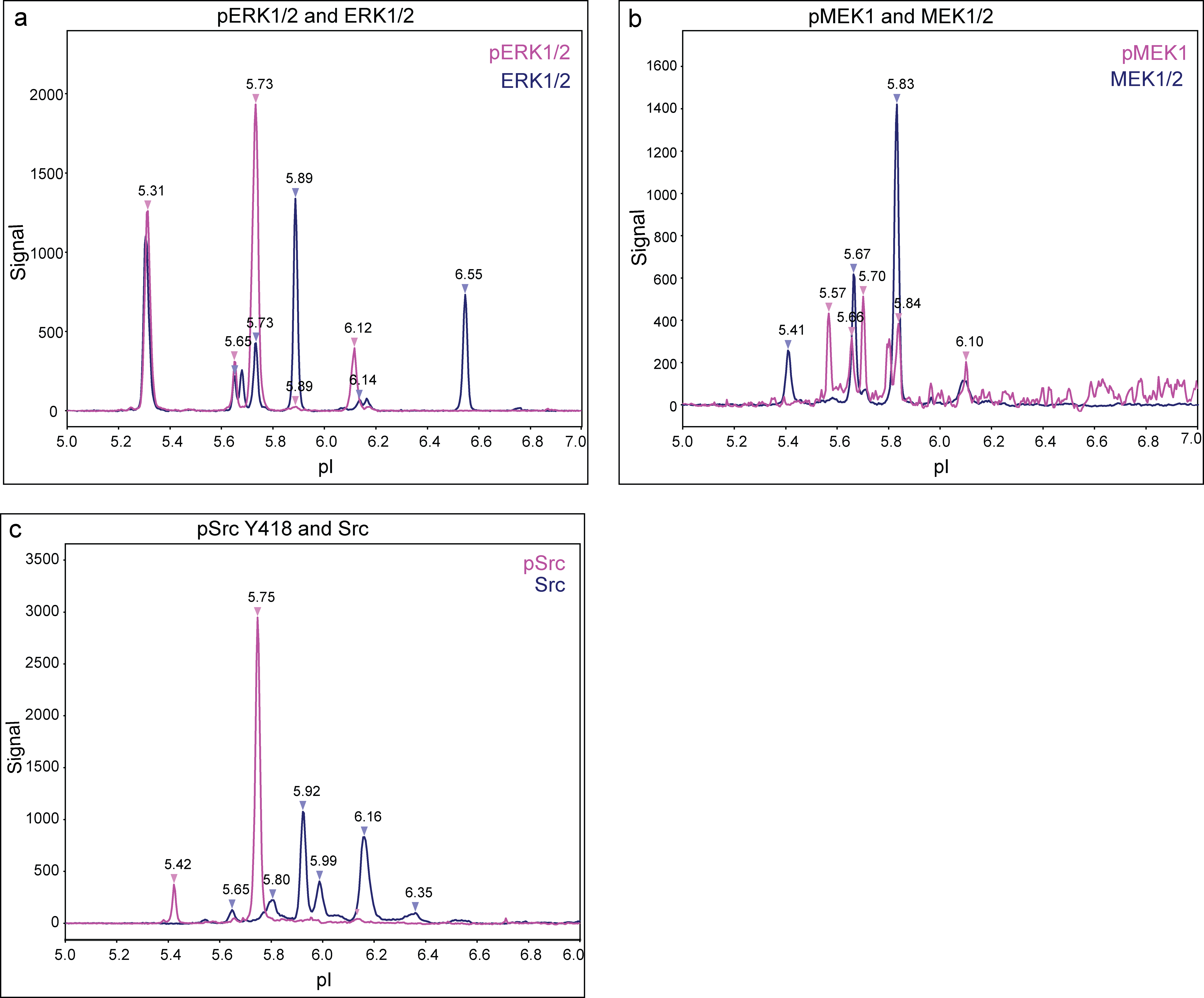
**

**Figure S4**. Representative electropherogram showing overlap of phospho and total protein peaks. NanoPro+RCA signals were collected using HUVEC cell lysate to show identify overlapping and unique peaks representing the different proteins. Typically, phosphoprotein variants appear as unique peaks with a more acidic pI than the total protein peaks. (a) Detailed overlay graph of pERK1/2 (pink) and ERK1/2 (blue) using 12.5 µg/ml cell lysate. Signals were from 30s exposure for both pERK1/2 and ERK1/2. (b) Detailed overlay graph of pMEK1 (pink) and MEK1/2 (blue) using 25 µg/ml cell lysate. Signals were from 480s exposure for pMEK1 and 60s exposure for MEK1/2. (c) Detailed overlay graph of pSrc Y418 (pink) and Src (blue) using 50 µg/ml cell lysate. Signals were from 30s exposure for both pSrc Y418 and total Src.


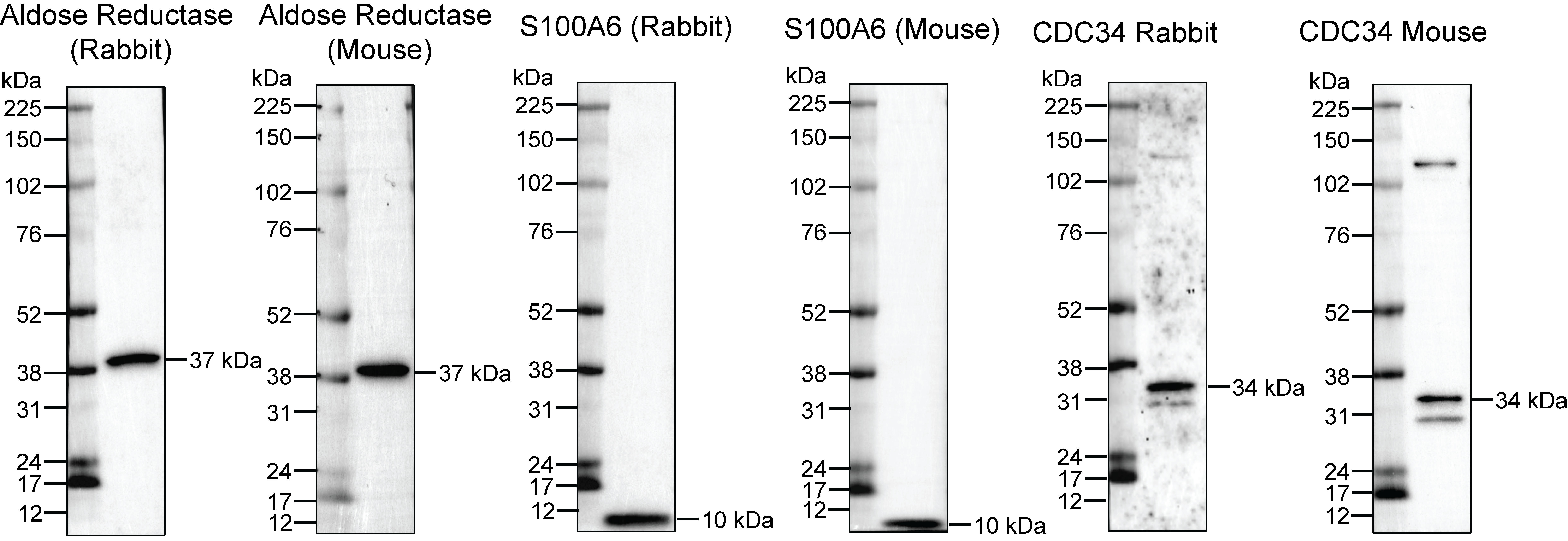


**Figure S5.** Antibody validation by immunoblotting. Images were acquired as described in Figure legend S1, using antibodies tested in the NanoPro+PLA analyses in main Figure 5. Ten µg of HUVEC cell lysate was used in each blot.

**PLA on NanoPro 1000 system with primary antibody-DNA conjugates**

The assays demonstrated in the study use secondary antibody-DNA conjugates. However, primary antibody-DNA conjugates can also be used with PLA and RCA. Here we demonstrate the technique and parameters to be optimized when using primary antibody-DNA conjugates in PLA for VEGFA detection.

For antibody-DNA conjugate preparations, two aliquots of 50 nM biotinylated anti-human VEGFA antibodies (BAF293, R&D systems) were mixed with 50 nM streptavidin-conjugated oligonucleotides Plus strand (Streptavidin-AAA AAA AAA ATA TGA CAG AAC TAG ACA CTC TT, Solulink) or with Minus strand (Streptavidin-AAA AAA AAA AGA CGC TAA TAG TTA AGA CGC TT, Solulink) in PBS with 0.1% bovine serum albumin (BSA) for 30 min at room temperature. After incubation, each of the two antibody-DNA complexes were diluted to 10 nM in PLA buffer (1 mM D-biotin (B1595, ThermoFisher Scientific), 0.1% BSA, 0.05% Tween 20, 100 nM goat IgG (I5256, Sigma-Aldrich), 0.1 μg/μl salmon sperm DNA (15632-011, ThermoFisher Scientific), 5 mM EDTA in PBS) and incubated at room temperature for 5 min. The antibody-DNA conjugates were then mixed, diluted to the appropriate concentrations in Antibody Diluent (#040-309, ProteinSimple), and used as probes in the assay. To establish suitable PLA parameters, 0.2 µg/ml recombinant human VEGFA (293-VE-010, R&D systems) was spiked in 50 µg/ml human dermal microcapillary endothelial cell (HDMEC) lysates, separated by capillary IEF, and detected with antibody-DNA conjugates (Figure S6). The ligation, RCA, and detection steps were the same as those described in Methods, except for the duration of the RCA. The signals for VEGFA detection increased with increasing probe concentrations, from 1.25 nM to 5 nM. Prolonging the RCA reaction time from 2 to 4 h resulted in a doubling of the signal, consistent with that RCA is a linear amplification reaction. The signal/noise (S/N) ratio extracted from the pI 8.4 VEGFA peak (Figure S7) increased with probe concentration and duration of RCA.

A parameter to consider for the hybridization and ligation step is the concentration of the two connector oligonucleotides that are joined into a DNA circle, templated by the antibody-DNA conjugates. The S/N ratios extracted from the pI 8.4 VEGFA peak analyzed at 0.02 µg/ml and 0.2 µg/ml, using oligonucleotide concentrations of 10 nM and 100 nM (Figure S8) were relatively stable. When back piece and connector oligonucleotide concentrations were reduced to 1 nM, the signal intensity dropped more than 50% as the hybridization and ligation efficiency decreased.

**Figure S6**. Illustration of PLA on NanoPro with primary antibody-DNA conjugates

**Figure S7.** Comparison of S/N ratios for titrations of probe concentrations and RCA time in capillary isoelectric focusing. RCA detection of 0.2 µg/ml recombinant VEGFA in 50 µg/ml HDMEC lysates using different probe concentration (5 nM, 2.5 nM and 1.25 nM) and RCA times (2 h and 4 h). Signal to noise was calculated for the pI 8.4 VEGFA peak. Error bars indicate standard deviations for duplicates.

**Figure S8.** S/N extracted for the pI 8.4 peak for VEGFA detection using different concentrations of connector oligonucleotides. Different concentrations of recombinant VEGFA in 50 µg/ml HDMEC lysates were used. Error bars indicate standard deviation for duplicate measurement.

**Table S1. Summary of primary antibodies data**

| Protein target | Species | Product # | Company | NanoPro,  NanoPro+RCA/  PLAdilution | Immunoblot dilution |
| --- | --- | --- | --- | --- | --- |
| p44/42 MAPK (ERK1/2) | Rabbit | 9102 | Cell Signaling | 1:100 | 1:1000 |
| Phospho-p44/42 MAPK (RK1/2) (Thr202/Tyr204) | Rabbit | 9101 | Cell Signaling | 1:50 | 1:1000 |
| Phospho-P38 | Rabbit | 4511 | Cell Signaling | 1:20 | 1:1000 |
| P38 | Mouse | 9228 | Cell Signaling | NA^3^ | 1:1000 |
| pMEK1 | Rabbit | ab32088 | Abcam | 1:30 | 1:1000 |
| MEK1/2 | Rabbit | sc-436 | Santa Cruz | NA | 1:1500 |
| β2 microglobulin | Rabbit | ab75853 | Abcam | 1:50 | 1:3000 |
| pSrcY418 | Rabbit | 44660G | Invitrogen | 1:50 | 1:1000 |
| Src | Rabbit | sc-18 | Santa Cruz | 1:40 | 1:1000 |
| Aldose reductase | Rabbit | HPA026425 | HPA^1^ | 1:50 | 1:1000 |
| Aldose reductase | Mouse | CPTC-AKR1B1-3 | NCI^2^ | 1:50 |
| S100A6 | Rabbit | HPA007575 | HPA | 1:50 | 1:1000 |
| S100A6 | Mouse | CPTC-Calcyclin-2 | NCI | 1:50 |
| CDC34 | Rabbit | HPA002382 | HPA | 1:50 | 1:1000 |
| CDC34 | Mouse | CPTC-CDC34-2 | NCI | 1:50 |
| HSP 70 | Mouse | NB600-571 | Novus Biologicals | 1:500 | 1:1000 |
| α-Tubulin | Mouse | T9026 | Sigma | NA | 1:1000 |

^1^HPA refers to Human Protein Atlas (<http://www.proteinatlas.org/>).

^2^NCI; National Cancer Institute (<http://www.cancer.gov/>).

^3^NA; not applicable.
